# Revisiting Epidermal Growth Factor Receptor (*EGFR*) Amplification as a Target for Anti-EGFR Therapy: Analysis of Cell-Free Circulating Tumor DNA in Patients With Advanced Malignancies

**DOI:** 10.1200/PO.18.00180

**Published:** 2019-01-22

**Authors:** Shumei Kato, Ryosuke Okamura, Manvita Mareboina, Suzanna Lee, Aaron Goodman, Sandip P. Patel, Paul T. Fanta, Richard B. Schwab, Peter Vu, Victoria M. Raymond, Richard B. Lanman, Jason K. Sicklick, Scott M. Lippman, Razelle Kurzrock

**Affiliations:** **Shumei Kato**, **Ryosuke Okamura**, **Manvita Mareboina**, **Suzanna Lee**, **Aaron Goodman**, **Sandip P. Patel**, **Paul T. Fanta**, **Richard B. Schwab**, **Peter Vu**, **Jason K. Sicklick**, **Scott M. Lippman**, and **Razelle Kurzrock**, University of California San Diego Moores Cancer Center, La Jolla; and **Victoria M. Raymond** and **Richard B. Lanman**, Guardant Health, Redwood City, CA.

## Abstract

**Purpose:**

To date, evidence for tissue epidermal growth factor receptor (EGFR) overexpression as a biomarker for anti-EGFR therapies has been weak. We investigated the genomic landscape of *EGFR* amplification in blood-derived cell-free tumor DNA (cfDNA) across diverse cancers and the role of anti-EGFR therapies in achieving response.

**Methods:**

We assessed *EGFR* amplification status among 28,584 patients with malignancies evaluated by clinical-grade next-generation sequencing (NGS) of blood-derived cfDNA (54- to 73-gene panel). Furthermore, we curated the clinical characteristics of 1,434 patients at the University of California San Diego who had cfDNA testing by this NGS test.

**Results:**

Overall, *EGFR* amplification was detected in cfDNA from 8.5% of patients (2,423 of 28,584), most commonly in colorectal (16.3% [458 of 2,807]), non–small-cell lung (9.0% [1,096 of 12,197]), and genitourinary cancers (8.1% [170 of 2,104]). Most patients had genomic coalterations (96.9% [95 of 98]), frequently involving genes affecting other tyrosine kinases (72.4% [71 of 98]), mitogen-activated protein kinase cascades (56.1% [55 of 98]), cell-cycle–associated signals (52.0% [51 of 98]), and the phosphoinositide 3-kinase pathway (35.7% [35 of 98]). *EGFR* amplification emerged in serial cfDNA after various anticancer therapies (n = 6), including checkpoint inhibitors (n = 4), suggesting a possible role for these amplifications in acquired resistance. Nine evaluable patients with *EGFR* amplification were treated with anti-EGFR–based regimens; five (55.6%) achieved partial responses, including three patients whose tissue NGS lacked *EGFR* amplification.

**Conclusion:**

*EGFR* amplification was detected in cfDNA among 8.5% of 28,584 diverse cancers. Most patients had coexisting alterations. Responses were observed in five of nine patients who received EGFR inhibitors. Incorporating EGFR inhibitors into the treatment regimens of patients harboring *EGFR* amplification in cfDNA merits additional study.

## INTRODUCTION

Epidermal growth factor receptor (EGFR), also known as human epidermal growth factor receptor 1 (HER1) or ErbB1, is a receptor tyrosine kinase that belongs to the ErbB family proteins. Along with EGFR, the ErbB family includes HER2 (ErbB2), HER3 (ErbB3), and HER4 (ErbB4). When receptor-specific ligands bind to the extracellular domain of the EGFR, it forms a homodimer (EGFR-EGFR) or heterodimer (eg, EGFR-HER2, EGFR-HER3) that leads to the activation of receptors through ATP-dependent phosphorylation of tyrosine residues in the EGFR intracellular domain. Activation of EGFR leads to multiple downstream signals, including mitogen-activated protein kinase and phosphoinositide 3-kinase pathways, which enhances cell proliferation and survival.^1,2^

Functional activation of EGFR via mutation or amplification/overexpression has been identified in many tumor types, including lung, head and neck, gastroesophageal, and colorectal cancers, and has been associated with proliferation, invasion, and metastasis.^3,4^ Alterations in *EGFR* have also been linked to primary resistance and accelerated tumor growth (designated as hyperprogression) from immune checkpoint inhibitors.^5-7^ Because of its critical role in tumor aggressiveness, EGFR has been an attractive target for anticancer therapy.^1^ To date, there are various anti-EGFR therapies that are US Food and Drug Administration approved, including erlotinib, gefitinib, afatinib, and osimertinib for non–small-cell lung cancer (NSCLC) with specific activating *EGFR* mutations,^8^ cetuximab and panitumumab for colorectal cancer without *KRAS* or *NRAS* mutations,^9^ cetuximab for head and neck cancer,^10^ and necitumumab for squamous cell carcinoma of lung.^11^

Biomarkers to predict response to anti-EGFR therapies have been studied extensively. *EGFR* and *KRAS* mutation status are widely used in lung and colorectal cancer, respectively.^8,9,12,13^ In contrast, *EGFR* amplification and overexpression in tissue have not been well established as reliable biomarkers for anti-EGFR agents, (selected studies that investigated *EGFR* amplification/overexpression as a predictive marker for anti-EGFR therapies are summarized in the Data Supplement)^11,14-19^. Overall, a meta-analysis concluded that tissue *EGFR* amplification status could not be demonstrated to be a consistent biomarker to predict the outcome from anti-EGFR therapies in colorectal cancer.^20^

Although it is somewhat surprising that tissue *EGFR* amplification/expression status has not been established as a reliable biomarker for anti-EGFR therapies, potential reasons include heterogeneity between primary and metastatic lesions, dynamic changes in genomic alterations that may emerge along with therapeutic pressure or progression, presence of genomic coalterations associated with resistance, and potential differences in response to copy number gain due to aneuploidy versus focal *EGFR* amplification.^21-23^ Use of plasma-derived cell-free tumor DNA (cfDNA) to assess *EGFR* status by next-generation sequencing (NGS) could conceivably overcome some of these limitations by detecting tumor-specific alterations that are shed into the bloodstream from multiple metastatic sites as well as the primary cancer.^23-29^

Herein, we examined the genomic landscape of *EGFR* amplification by interrogating blood-derived cfDNA from 28,584 patients with diverse malignancies using clinical-grade NGS. Furthermore, we investigated the clinical characteristics, concordance between tissue NGS and cfDNA, and therapeutic outcome after anti-EGFR therapies among a subset of 1,434 clinically annotated patients at the University of California, San Diego (UCSD), Moores Cancer Center.

## METHODS

### Patients

The genomic landscape of *EGFR* amplification among 28,584 diverse solid cancers that were referred to Guardant Health from March 2014 to February 2017, were evaluated. Furthermore, we have curated the clinical characteristics of 1,434 evaluable patients with diverse cancers at UCSD who had cfDNA testing at Guardant Health starting in March 2014. All investigations followed the guidelines of the UCSD Institutional Review Board for data collection (Profile Related Evidence Determining Individualized Cancer Therapy; ClinicalTrials.gov identifier: NCT02478931) and for any investigational therapies for which the patients consented (Data Supplement).

### NGS for cfDNA and Tissue

All cfDNA analyses were performed at Guardant Health as previously described (Data Supplement).^26^ Tissue NGS was performed at Foundation Medicine, as previously described^30^ (Data Supplement).

### End Points and Statistical Methods

Patient characteristics, prevalence of *EGFR* amplification, and genomic coalterations were summarized by descriptive statistics. Fisher’s exact test was used for categorical variables. Concordances between cfDNA and tissue DNA were described by percentage of concordance and κ value with standard error. Antitumor response was evaluated using Response Evaluation Criteria in Solid Tumors (RECIST) 1.1. Progression-free survival was defined as the time from treatment initiation to disease progression. Patients who had not experienced disease progression at the time of last follow-up were censored at that time point. Statistical analysis was performed with the assistance of author R.O.

## RESULTS

### Prevalence of *EGFR* Amplification in cfDNA Testing in Diverse Cancers

**Fig 1.**
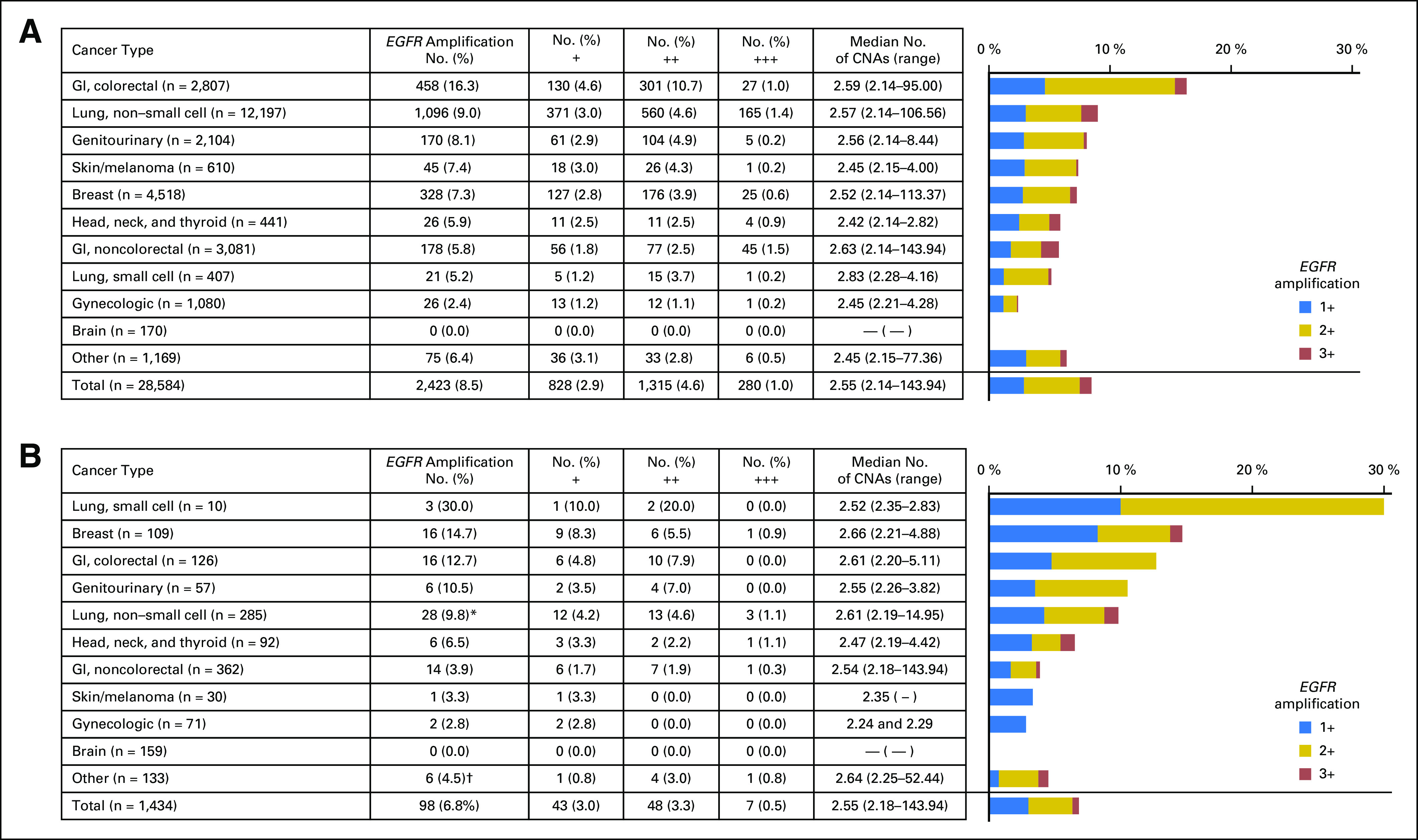
(A) Prevalence of *EGFR* amplification by cell-free DNA (cfDNA) among diverse cancer from central laboratory (n = 28,584). Among 28,584 patients with diverse cancer whose cfDNA was evaluated at central laboratory, overall 8.5% of patients (n = 2,423) had *EGFR* amplification (median copy number amplification [CNA], 2.55; range, 2.14 to 143.94). Overall, 1+, 2+, and 3+ *EGFR* CNAs were found in 2.9% (n = 828), 4.6% (n = 1,315), and 1.0% (n = 280) of patients, respectively. The most common cancer harboring *EGFR* amplification was colorectal cancer (16.3% [458 of 2,807]), followed by non–small-cell lung cancer (9.0% [1,096 of 12,197]) and genitourinary cancers (8.1% [170 of 2,104]). (B) Prevalence of *EGFR* amplification by cfDNA among diverse cancer from University of California, San Diego (UCSD), cohort (n = 1,434). Among UCSD cohort (n = 1,434), overall 6.8% of patients (98 of 1,434) had *EGFR* amplification (median CNA, 2.55; range, 2.18 to 143.94). CNAs of 1+, 2+, and 3+ were found in 3.0% (n = 43), 3.3% (n = 48), and 0.5% (n = 7) of patients, respectively. The most common cancer harboring *EGFR* amplification was small-cell lung cancer (30.0% [three of 10]), followed by breast cancer (14.7% [16 of 109]) and colorectal cancer (12.6% [16 of 127]). *Includes 25 patients with lung, adenocarcinoma (+ [n = 10], ++ [n = 12], +++ [n = 3]) and three patients with lung, squamous cell carcinoma (+ [n = 2], ++ [n = 1]). †Includes four patients with carcinoma of unknown primary (+ [n = 1], ++ [n = 2], +++ [n = 1]) and two patients with adrenocortical carcinoma (++ [n = 2]).

Among 28,584 patients with diverse solid malignancies whose cfDNA was evaluated at a central laboratory, 8.5% (n = 2,423) had *EGFR* amplification. The most common tumors harboring *EGFR* amplification were colorectal cancer (16.3% [458 of 2,807]), followed by NSCLC (9.0% [1,096 of 12,197]), genitourinary cancers (8.1% [170 of 2,104]), cutaneous tumors (7.4% [45 of 610]), and breast cancer (7.3% [328 of 4,518]; Fig 1A).

### Prevalence of *EGFR* Amplification in cfDNA Testing in Patients With Diverse Cancers From UCSD Cohort

Among the UCSD cohort of 1,434 patients (Data Supplement), overall, 6.8% of patients (98 of 1,434) had *EGFR* amplification, including 86 patients detected at their first cfDNA evaluation and 12 patients with emerging *EGFR* amplification at the time of subsequent cfDNA evaluation. The most common cancers (with more than 10 samples) harboring *EGFR* amplification were breast (14.7% [16 of 109]) and colorectal cancer (12.7% [16 of 126]; Fig 1B).

### Genomic Coalterations Associated With *EGFR* Amplification (analysis of cfDNA)

**Fig 2.**
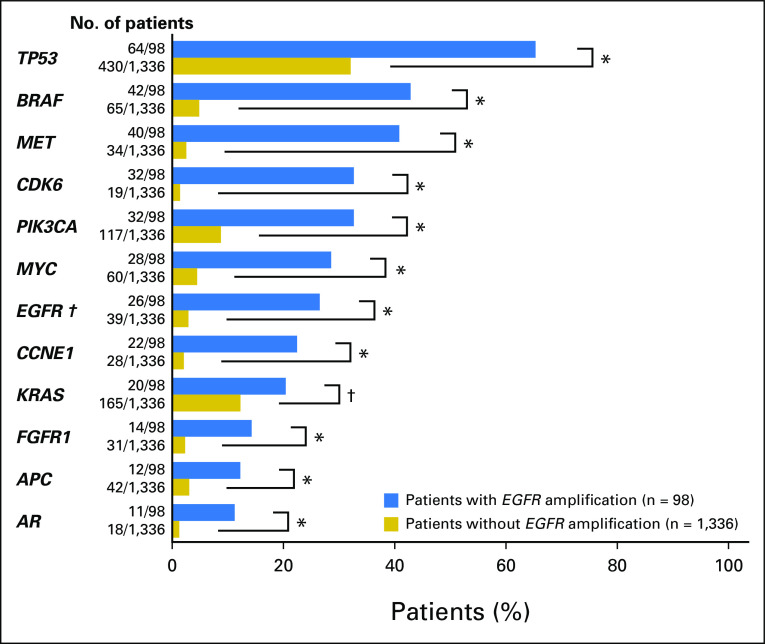
Comparison of genomic alterations in patients with and without *EGFR* amplification (analysis of cell-free DNA [cfDNA]; n = 1,434). The most common cogenomic alterations associated with *EGFR* amplification was *TP53* (65.3% [64 of 98]), followed by *BRAF* (42.9% [42 of 98]), *MET* (40.8% [40 of 98]), *CDK6* (32.7% [32 of 98]), and *PIK3CA* (32.7% [32 of 98]). On the other hand, coalterations in these genes were significantly less associated among patients without *EGFR* amplification (alterations found: 32.1% in *TP53*, 4.9% in *BRAF*, 2.5% in *MET*, 1.4% in *CDK6*, and 8.8% in *PIK3CA*; all *P* < .001). *P < .001. **P = .027 by Fisher’s exact test. †EGFR alterations other than amplification. Variants of unknown significance excluded.

Among 98 patients with *EGFR* amplification at UCSD, the median number of characterized genomic alterations was 5.0 (range, 0 to 17; excluding the *EGFR* amplification), and the median number of alterations among patients without *EGFR* amplification (n = 1,336) was significantly less (median, 1.0; range, 0 to 20; *P* < .001). The most common coalterations associated with *EGFR* amplification were in the following genes: *TP53* (65.3% [64 of 98]), followed by *BRAF* (42.9% [42 of 98]), *MET* (40.8% [40 of 98]), *CDK6* (32.7% [32 of 98]), and *PIK3CA* (32.7% [32 of 98]; Fig 2; Appendix Fig A1). On the other hand, coalterations in these genes were found significantly less frequently among patients without *EGFR* amplification: coalterations in *TP53* in 32.1% of patients, *BRAF* (4.9%), *MET* (2.5%), *CDK6* (1.4%), and *PIK3CA* (8.8%; all *P* < .001; Fig 2). When the genes were categorized according to their oncogenic roles, 72.4% (71 of 98) of patients with *EGFR* amplification had at least one characterized coalteration in tyrosine kinase family genes, 56.1% (55 of 98) in genes involved in mitogen-activated protein kinase cascades, 52.0% (51 of 98) in cell-cycle–associated genes, and 35.7% (35 of 98) in phosphoinositide 3-kinase signaling pathway genes (Appendix Fig A2).

### Potential Targeted Therapies for Coalterations Associated With *EGFR* Amplification

In the UCSD cohort of 98 patients positive for *EGFR* amplification, 96.9% (95 of 98) of patient tumors had at least one characterized coalteration. All these 95 malignancies harbored at least one characterized coalteration potentially targetable with US Food and Drug Administration–approved agents as on- or off-label use.

### Clinical Characteristics of Patients Who Had Emerging *EGFR* Amplification With Serial cfDNA Analyses

**Fig 3.**
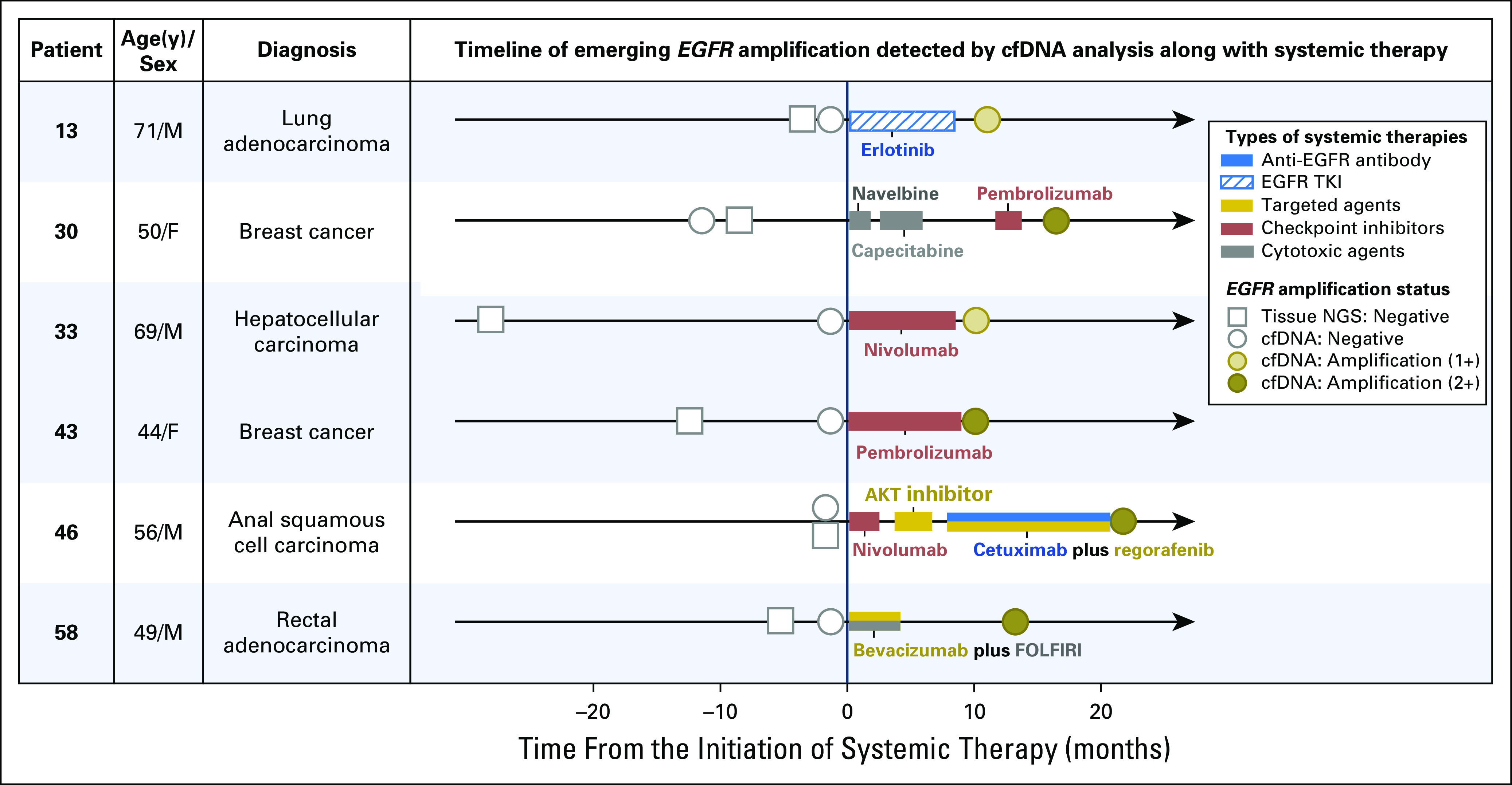
Overview of patients who had emerging *EGFR* amplification with serial cell-free DNA (cfDNA) analysis after anticancer therapies (Data Supplement). Six patients who initially tested negative for *EGFR* amplification on tissue next-generation sequencing as well as cfDNA were found to have *EGFR* amplification with serial cfDNA analyses after various treatments; in four patients, treatment regimens included immune checkpoint inhibitors (n = 4; patients 30, 33, 43, and 46). EGFR, epidermal growth factor receptor; FOLFIRI, fluorouracil, leucovorin, and irinotecan; NGS, next-generation sequencing; TKI, tyrosine kinase inhibitor.

Six patients who initially tested negative for *EGFR* amplification in both tissue NGS and cfDNA were found to have emerging *EGFR* amplification with serial cfDNA analyses. (A total of 324 patients who initially tested negative had serial testing.) Patterns of genomic evolution differed from patient to patient. Emergence of *EGFR* amplification was seen among patients in a variety of situations, including in four patients who received checkpoint inhibitors, although two of the four patients also had other intervening therapies (Fig 3; Data Supplement).

### Concordance of *EGFR* Amplification Between cfDNA and Tissue NGS

Among patients from the UCSD cohort whose cfDNA was evaluated, tissue NGS was available in 787 cases. The overall concordance rate for *EGFR* amplification between tissue and cfDNA NGS was 89.3% (Data Supplement). A shorter interval between the date of tissue biopsy (for tissue NGS) and blood draw (for cfDNA) was associated with statistically higher concordance rate (≤ 6 months: 92.1% *v* > 6 months: 85.8%; *P* = .005; Data Supplement).

### Efficacy of Anti-EGFR Therapies Among Patients With *EGFR* Amplification Detected by cfDNA Analysis

**Fig 4.**
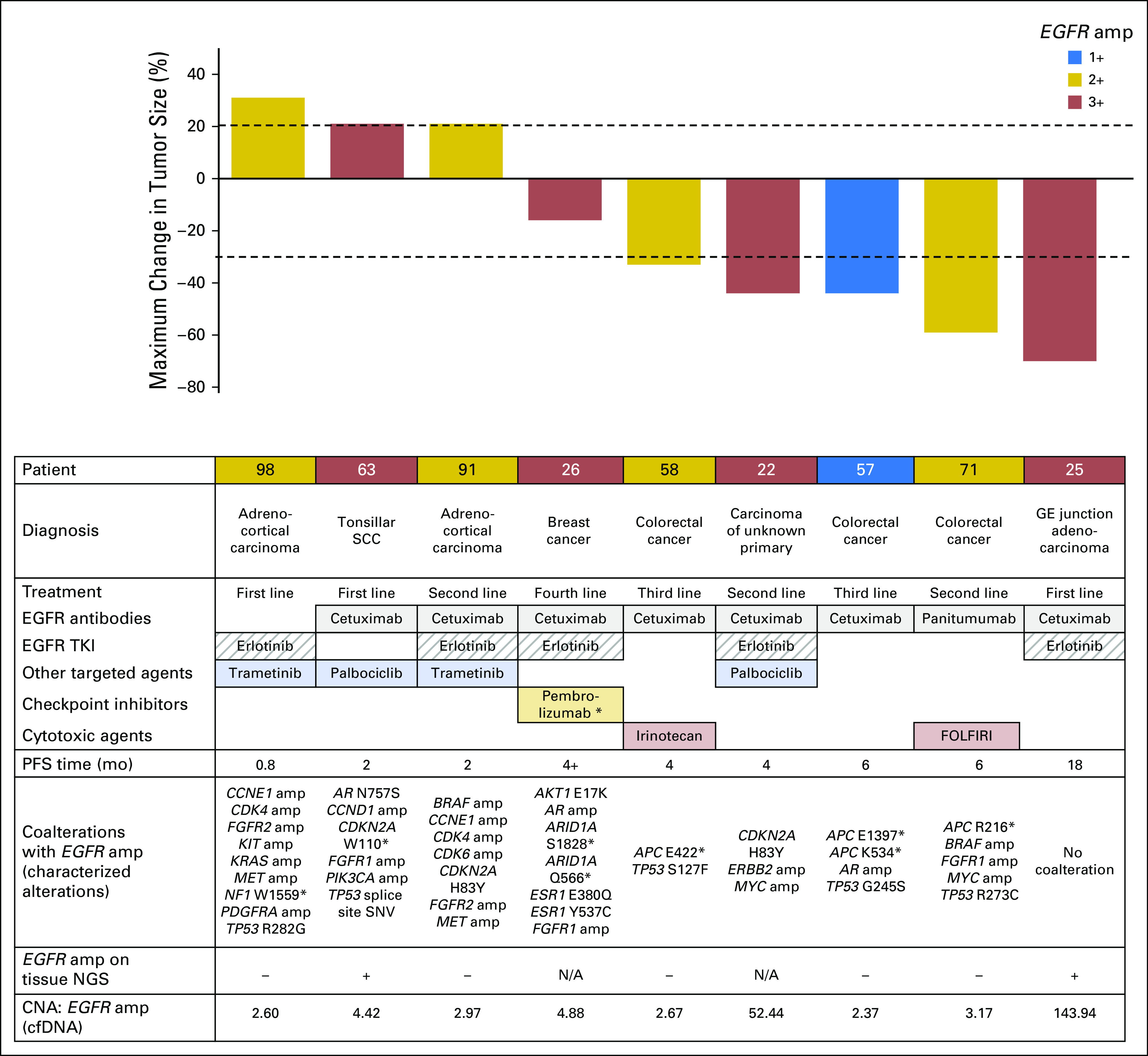
Efficacy of anti–epidermal growth factor receptor (EGFR)–based therapies among patients with *EGFR* amplification (amp). Nine patients with *EGFR* amplification were evaluable for response from anti-EGFR–based therapies. Overall, tumor reduction was seen in six of nine (66.7%), including five of nine (55.6%) patients with partial response per Response Evaluation Criteria in Solid Tumors 1.1. (*) Patient was experiencing mixed response (mixture of disease stability and progressive disease) with pembrolizumab monotherapy after 15 months. Anti-EGFR agents were added for emerging EGFR amplification detected in cfDNA (see also Figure 5). cfDNA, cell-free DNA; CNA, copy number amplification; EGFR, epidermal growth factor receptor; FOLFIRI, fluorouracil, leucovorin, and irinotecan; GE, gastroesophageal; N/A, not applicable; NGS, next-generation sequencing; PFS, progression-free survival; SCC, squamous cell carcinoma; TKI, tyrosine kinase inhibitor.

**Fig 5.**
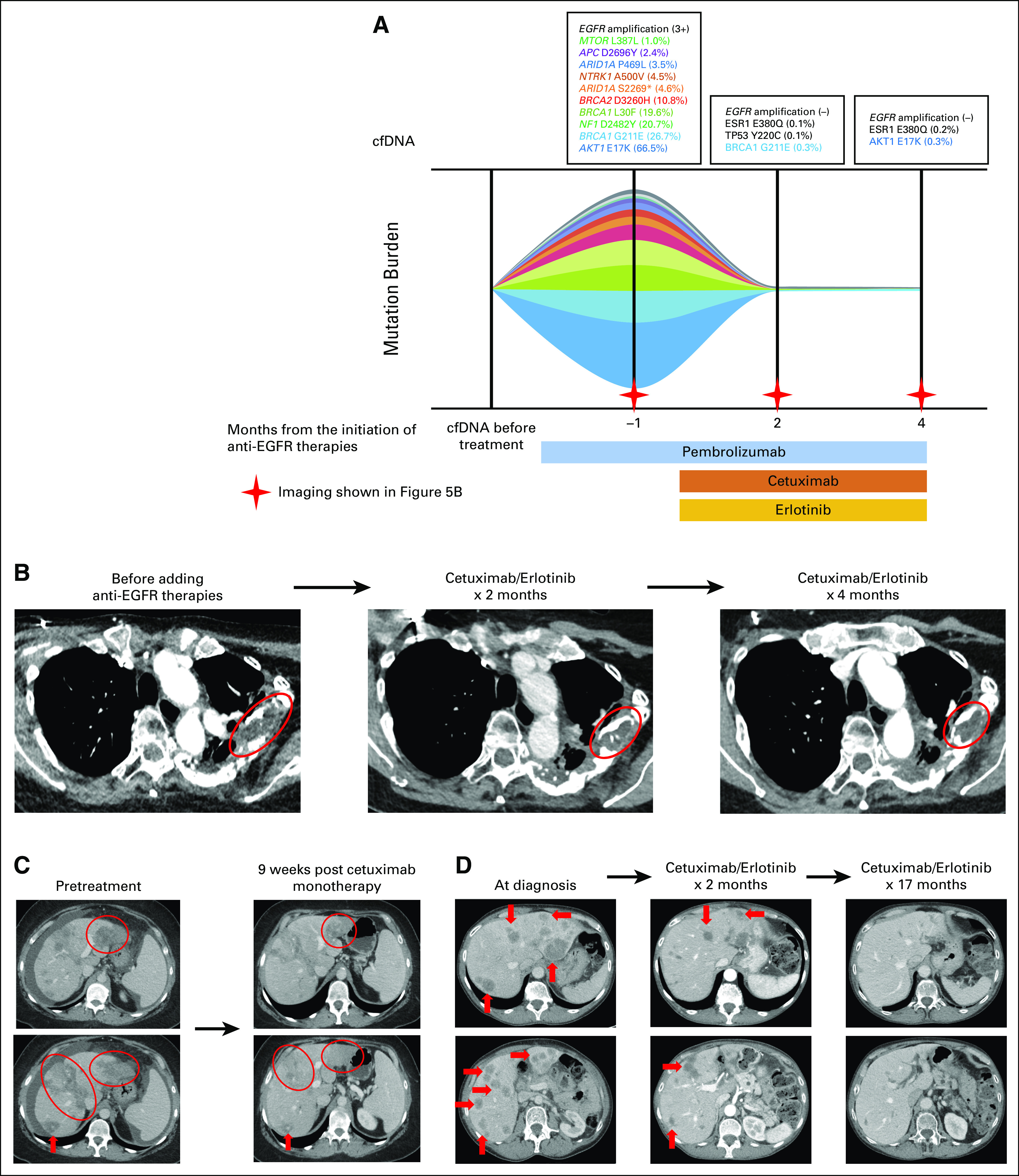
Representative cases of patients who achieved tumor reduction with anti–epidermal growth factor receptor (EGFR)–based therapies. (A) Patient 26: Dynamic change in serial cell-free DNA (cfDNA) along with anti-EGFR therapies. (B) Patient 26: Serial computed tomography (CT) images while receiving anti-EGFR therapies (correspond with A). A 55-year-old woman with metastatic triple-negative breast cancer to bone and lung was treated with pembrolizumab with initial disease stability for more than 1 year, but then progression in the bones and deteriorating performance status requiring a wheelchair. cfDNA obtained before treatment did not detect genomic alterations. cfDNA at the time of bone progression revealed multiple emerging alterations, including *EGFR* amplification (3+; plasma copy number of 4.88; A). Pembrolizumab was continued and anti-EGFR therapies (erlotinib and cetuximab) were added. After starting anti-EGFR agents, the patient achieved 16% tumor shrinkage per Response Evaluation Criteria in Solid Tumors (RECIST) 1.1 (B, left to right), with symptomatic improvement allowing the patient to ambulate without narcotics for pain control. Repeat cfDNA showed elimination of most alterations, including *EGFR* amplification (A). Therapy with anti-EGFR inhibitors is ongoing at 4+ months. (C) Patient 57: Serial CT images while receiving anti-EGFR therapy. A 53-year-old woman with metastatic rectal adenocarcinoma to the liver and lungs presented after experiencing disease progression while receiving two lines of therapies (infusional fluorouracil, leucovorin, and oxaliplatin with bevacizumab and fluorouracil, leucovorin, and irinotecan with bevacizumab). Tissue next-generation sequencing was negative for *EGFR* amplification; however, cfDNA revealed alterations including *EGFR* amplification (1+; plasma copy number of 2.37). Therapy was started with single-agent cetuximab, and a 44% reduction in tumor burden by RECIST 1.1 was seen (progression-free survival, 6.0 months; C, left to right). (D) Patient 25: Serial CT images while receiving anti-EGFR therapies. A 68-year-old woman was referred with metastatic gastroesophageal junction carcinoma to the liver and lymph nodes. Both cfDNA and primary tumor showed *EGFR* amplification by next-generation sequencing (*EGFR* amplification 3+ by cfDNA, plasma copy number of 143.94). Therapy with dual anti-EGFR therapy (cetuximab and erlotinib) was started (patient was also administered one dose of nivolumab on the basis of programmed death ligand 1 positive by immunohistochemistry; however, held because of severe rash). The patient achieved a durable partial response^28^ (70% tumor reduction; progression-free survival, 18 months; D, left to right). Serial cfDNA analyses were obtained at 4 months, 12 months, and 17 months after the initiation of therapy and were negative for *EGFR* amplification. Red arrows and circles indicate presence of tumor. cfDNA, cell-free DNA.

Among 98 patients with *EGFR* amplification, patients harboring coactivating *EGFR* mutations were excluded from the analysis, because the response from anti-EGFR therapies could be confounded by these mutations (n = 26). Among 72 patients with *EGFR* amplification (without coexisting *EGFR* mutations), nine received treatment regimens that included anti-EGFR agents after cfDNA testing (Appendix Fig A3). Among these nine individuals, *EGFR* plasma copy numbers ranged from 2.37 to 143.94 (1+ [n = 1], 2+ [n = 4], and 3+ [n = 4]) across six different cancer diagnoses: tonsillar squamous cell carcinoma (n = 1), triple-negative breast cancer (n = 1), adenocarcinoma of unknown primary (n = 1), gastroesophageal junction adenocarcinoma (n = 1), adrenocortical carcinoma (n = 2), and colorectal cancer (n = 3). Types of anti-EGFR–based regimens were as follows: monotherapy with anti-EGFR antibody (n = 1), anti-EGFR antibody plus another targeted agent (n = 1), EGFR tyrosine kinase inhibitor plus another targeted agent (n = 1), anti-EGFR antibody plus cytotoxic agents (n = 2), and dual anti-EGFR therapy–based regimens (combination of anti-EGFR antibody plus EGFR tyrosine kinase inhibitor; n = 4). Overall, tumor reduction was seen in six of nine patients (66.7%), including five (55.6%) who attained a partial response (PR) per RECIST 1.1 (Fig 4**)**. Illustrative responders are depicted in Figure 5.^28^

## DISCUSSION

We describe the comprehensive landscape of *EGFR* amplification in cfDNA among 28,584 patients with varied malignancies whose liquid biopsy was evaluated at a central, clinical-grade laboratory. Overall, 8.5% of patients harbored an *EGFR* amplification in their blood-derived cfDNA, with *EGFR* amplifications being most common in colorectal cancer (16.3% of patients), NSCLC (9.0%), genitourinary cancers (8.1%), cutaneous tumors (7.4%), and breast malignancies (7.3%). Having high copy number amplification with greater than 4.00-fold *EGFR* amplification was seen in 1.0% of patients; most patients had amplification levels between 2.41-fold and 4.00-fold (4.6%; Fig 1).

*EGFR* amplification/overexpression is associated with cancer aggressiveness.^3,4^ Even so, previous studies failed to demonstrate tissue-based assessment of *EGFR* overexpression to be a reliable biomarker to predict clinical outcomes after anti-EGFR therapies (Data Supplement).^11,14-20^ These observations are consistent with data from meta-analyses and clinical experience suggesting that, counterintuitively, genomic biomarkers correlate better with response than protein expression, perhaps because of technical limitations associated with assessment of immunohistochemistry staining.^31-34^

Prior studies looking at the relationship between *EGFR* amplification and therapeutic response to EGFR inhibitors showed inconsistent results (Data Supplement). However, one potential explanation is tumor heterogeneity, especially between primary and metastatic lesions and even between distinct foci at the same site. Indeed, Pectasides et al^23^ demonstrated that, among patients with treatment-naive metastatic gastroesophageal cancers, discordant gene alterations between primary and metastatic tissue were common, being seen in 42% of patients. Interestingly, the discordance rate was higher for gene amplifications. However, among discordant cases, high concordance (87.5%) was seen between metastatic tissue and cfDNA profiling. The concordance rate documented by Pectasides et al^23^ is similar to that in the current report that showed an 89.3% concordance rate for *EGFR* amplification between cfDNA and tissue NGS (Data Supplement). These results suggest that biopsy of a limited tumor focus can misrepresent the overall genomic condition of disease and, thus, may not be a completely accurate guide for targeted treatment. NGS of cfDNA derived from plasma may attenuate this challenge. Consistent with this concept, among our nine evaluable patients who harbored *EGFR* amplification by cfDNA analysis, anti-EGFR–based therapies led to tumor reduction in 66.7% (six of nine) including 55.6% (five of nine) who achieved a PR (Fig 4). Our data are comparable to those of Maron et al,^35^ who showed a 58% (four of seven) objective response rate among patients with *EGFR*-amplified gastric cancer (all seven patients were positive for *EGFR* amplification by tissue NGS, and six were positive by cfDNA analysis). Similarly, *ERBB2* amplification detected by cfDNA analysis was highly predictive of anti-HER2 targeted therapy response.^36^

There were additional noteworthy observations from our patients treated with anti-EGFR–based therapies: three of five patients whose *EGFR* amplification was only detected in cfDNA (negative on tissue NGS) still demonstrated a PR (including one patient treated with cetuximab monotherapy [patient 57]), PRs were seen across different degrees of *EGFR* amplification status (from copy number amplification of 2.37 to 143.94), and three of four patients who received dual anti-EGFR inhibitors (coadministration of antibody and tyrosine kinase inhibitor for EGFR) achieved tumor reduction. Importantly, dual inhibition with both an antibody and a small molecule targeting the same receptor has been investigated among patients with HER2-positive breast cancer and reported to have significantly higher response rates when compared with either drug alone.^37^ Efficacy of dual-targeted therapy was also seen in patients with HER2-positive colon cancer that showed a 30% response rate with trastuzumab/lapatinib combination.^38^ Similarly, early-phase clinical trials with dual-EGFR inhibition (cetuximab/afatinib- or cetuximab/erlotinib-based therapy) showed favorable clinical outcomes among patients with refractory NSCLC and colorectal cancer.^39-43^ The mechanism by which dual inhibition operates is not fully elucidated, but preclinical studies suggest that kinase receptors may function via kinase-dependent and -independent mechanisms.^44,45^

Although responses were seen in more than half of the patients with *EGFR* amplification treated with EGFR inhibitors, not all patients responded. Our study demonstrates that patients whose tumors harbor *EGFR* amplifications have considerably more cfDNA genomic alterations than those without *EGFR* amplification (median, 5.0 *v* 1.0 genomic coalteration per patient; *P* < .001). Therefore, primary or secondary resistance could be on the basis of the need to target coexisting activated pathways. Indeed, as seen in Figure 4, of the nine patients with cfDNA *EGFR* amplification treated with EGFR-targeting agents, the four nonresponders had six to nine genomic coalterations, whereas the five responders had only zero to five coalterations per patient. Furthermore, the patient with the greatest tumor regression and most durable response (Fig 4, patient 25; progression-free survival, 18 months) demonstrated no genomic coalterations on cfDNA. Of interest, patients who failed to achieve prolonged responses had coalterations in specific oncogenic pathways, including *CDK4/6*, *MET*, *PDGFRA*, *ERBB2*, *FGFR1*, *PIK3CA*, AKT1, *KRAS*, and *BRAF*, some of which are known to be associated with resistance to anti-EGFR therapies.^46,47^ Considering that patients with *EGFR* amplification had frequent potentially tractable coalterations (Fig 2; Appendix Figs A1 and A2), a customized combination strategy may be required.^48,49^ Although current findings do not provide definitive proof of antitumor activity, these observations suggest that studies of appropriate combinations of drugs that target both the *EGFR* amplification and the coalterations would be of interest. Investigation of such an approach is currently ongoing (ClinicalTrial.gov identifier: NCT02534675; I-PREDICT [Study of Molecular Profile-Related Evidence to Determine Individualized Therapy for Advanced or Poor Prognosis Cancers]).

Interestingly, Oxnard et al^50^ and Abbosh et al^51^ have shown that more extensive disease burden corresponds to higher rates of cfDNA detection. The finding of higher numbers of comutations in patients harboring *EGFR* amplification events could therefore be a possible effect of increased aggressiveness and higher tumor burden (with more extensive disease shedding more cfDNA and thus permitting detection of more alterations), or, alternatively, higher numbers of comutations could be a cause of increased aggressiveness. In this regard, we have recently found that higher percent cfDNA correlates with higher number of alterations. Furthermore, both higher percent cfDNA and higher number of alterations were independently associated with shorter survival after multivariate analysis. This observation suggests that the association between survival and number of alterations is independent of the percent cfDNA (with the latter correlating with disease burden; unpublished data).

In the current report, we also identified patients whose *EGFR* amplifications emerged in their liquid biopsy with serial testing after a variety of anticancer therapies (n = 6; Fig 3). For instance, one patient who was treated with the EGFR tyrosine kinase inhibitor erlotinib showed emergence of blood-derived *EGFR* amplification after disease progression (Fig 4), consistent with a previous report demonstrating tumor evolution with *EGFR* amplification as a potential resistance mechanism to EGFR tyrosine kinase inhibitor administration.^52^ Perhaps relevant in this regard, all of our responders had an EGFR antibody included in their regimen. Four patients were found to have emerging *EGFR* amplification after disease progression while receiving checkpoint inhibitors. Although *EGFR* alterations are reported to be associated with primary resistance and hyperprogression after immune checkpoint blockade,^6,7^ the current observation may suggest that *EGFR* amplification can also be a possible mechanism for acquired resistance after checkpoint blockades. For those patients with clonal evolution that includes *EGFR* amplification after anti–PD-1 checkpoint blockade, addition of anti-EGFR therapy may overcome resistance. This is suggested by our representative patient who was treated with pembrolizumab, had a mixed response, and then received erlotinib and cetuximab (in addition to ongoing pembrolizumab) and showed reduction in *EGFR* cfDNA copy number as well as regression of tumor foci and improvement in pain and performance status (Fig 4, patient 26; Figs 5A and 5B).^39,40^ Additional investigation is required to understand the complex interplay of response and resistance associated with *EGFR* amplifications, EGFR-targeting pharmaceuticals, and checkpoint blockade.

There were several limitations to the current study. First, the investigation of clinical correlates in the UCSD cohort was performed retrospectively. Second, for the large de-identified database of 28,584 patients, sample size bias cannot be excluded, because the number of each cancer type was based on the number of samples sent for cfDNA testing by treating physicians. Moreover, the diagnosis was determined based on the submitting physician’s designation. Third, in the de-identified database, we were not able to evaluate the history of systemic therapy that may have affected the dynamics of cfDNA. It is possible that some of the *EGFR* amplifications emerged because of therapeutic pressure. Last, technological methods and definition of *EGFR* amplification differed between tissue NGS and cfDNA analyses; thus, direct comparison between those two tests may be challenging. Yet, despite these limitations, the study provides a comprehensive analysis of *EGFR* amplification detected from plasma-derived cfDNA in a wide range of malignancies.

In conclusion, among patients with diverse cancers (n = 28,584 from a central laboratory), cfDNA interrogated by clinical-grade NGS revealed that 8.5% of patients with solid cancers harbored *EGFR* amplification. Frequencies of *EGFR* amplification differed between cancer types. Most patients found to have *EGFR* amplification also had genomic coalterations that are, in theory, pharmacologically tractable (96.9% [95 of 98]) by available drugs. Anti-EGFR–based therapies among patients found to have *EGFR* amplification by cfDNA analysis achieved responses in 55.6% of patients (five of nine), including in three individuals who failed to show *EGFR* amplification on tissue NGS. Incorporating EGFR inhibitors into regimens administered to patients with *EGFR* amplification in cfDNA warrants additional investigation.

## References

[1] Ciardiello F Tortora G EGFR antagonists in cancer treatment N Engl J Med 358 1160 1174 2008 18337605 10.1056/NEJMra0707704

[2] Normanno N De Luca A Bianco C et al Epidermal growth factor receptor (EGFR) signaling in cancer Gene 366 2 16 2006 16377102 10.1016/j.gene.2005.10.018

[3] Citri A Yarden Y EGF-ERBB signalling: Towards the systems level Nat Rev Mol Cell Biol 7 505 516 2006 16829981 10.1038/nrm1962

[4] Lu Z Jiang G Blume-Jensen P et al Epidermal growth factor-induced tumor cell invasion and metastasis initiated by dephosphorylation and downregulation of focal adhesion kinase Mol Cell Biol 21 4016 4031 2001 11359909 10.1128/MCB.21.12.4016-4031.2001 PMC87064

[5] Champiat S Dercle L Ammari S et al Hyperprogressive disease is a new pattern of progression in cancer patients treated by anti-PD-1/PD-L1 Clin Cancer Res 23 1920 1928 2017 27827313 10.1158/1078-0432.CCR-16-1741

[6] Kato S Goodman A Walavalkar V et al Hyperprogressors after immunotherapy: Analysis of genomic alterations associated with accelerated growth rate Clin Cancer Res 23 4242 4250 2017 28351930 10.1158/1078-0432.CCR-16-3133 PMC5647162

[7] Rizvi H Sanchez-Vega F La K et al Molecular determinants of response to anti-programmed cell death (PD)-1 and anti-programmed death-ligand 1 (PD-L1) blockade in patients with non-small-cell lung cancer profiled with targeted next-generation sequencing J Clin Oncol 36 633 641 2018 29337640 10.1200/JCO.2017.75.3384 PMC6075848

[8] Tan CS Gilligan D Pacey S Treatment approaches for EGFR-inhibitor-resistant patients with non-small-cell lung cancer Lancet Oncol 16 e447 e459 2015 26370354 10.1016/S1470-2045(15)00246-6

[9] Allegra CJ Jessup JM Somerfield MR et al American Society of Clinical Oncology provisional clinical opinion: Testing for KRAS gene mutations in patients with metastatic colorectal carcinoma to predict response to anti-epidermal growth factor receptor monoclonal antibody therapy J Clin Oncol 27 2091 2096 2009 19188670 10.1200/JCO.2009.21.9170

[10] Vermorken JB Mesia R Rivera F et al Platinum-based chemotherapy plus cetuximab in head and neck cancer N Engl J Med 359 1116 1127 2008 18784101 10.1056/NEJMoa0802656

[11] Thatcher N Hirsch FR Luft AV et al Necitumumab plus gemcitabine and cisplatin versus gemcitabine and cisplatin alone as first-line therapy in patients with stage IV squamous non-small-cell lung cancer (SQUIRE): An open-label, randomised, controlled phase 3 trial Lancet Oncol 16 763 774 2015 26045340 10.1016/S1470-2045(15)00021-2

[12] Tsigelny IF Wheler JJ Greenberg JP et al Molecular determinants of drug-specific sensitivity for epidermal growth factor receptor (EGFR) exon 19 and 20 mutants in non-small cell lung cancer Oncotarget 6 6029 6039 2015 25760241 10.18632/oncotarget.3472 PMC4467419

[13] Wheler JJ Falchook GS Tsimberidou AM et al Aberrations in the epidermal growth factor receptor gene in 958 patients with diverse advanced tumors: Implications for therapy Ann Oncol 24 838 842 2013 23139256 10.1093/annonc/mds524 PMC4110484

[14] Bokemeyer C Bondarenko I Hartmann JT et al Efficacy according to biomarker status of cetuximab plus FOLFOX-4 as first-line treatment for metastatic colorectal cancer: The OPUS study Ann Oncol 22 1535 1546 2011 21228335 10.1093/annonc/mdq632

[15] Cappuzzo F Finocchiaro G Rossi E et al EGFR FISH assay predicts for response to cetuximab in chemotherapy refractory colorectal cancer patients Ann Oncol 19 717 723 2008 17974556 10.1093/annonc/mdm492

[16] Dragovich T McCoy S Fenoglio-Preiser CM et al Phase II trial of erlotinib in gastroesophageal junction and gastric adenocarcinomas: SWOG 0127 J Clin Oncol 24 4922 4927 2006 17050876 10.1200/JCO.2006.07.1316

[17] Herbst RS Redman MW Kim ES et al Cetuximab plus carboplatin and paclitaxel with or without bevacizumab versus carboplatin and paclitaxel with or without bevacizumab in advanced NSCLC (SWOG S0819): A randomised, phase 3 study Lancet Oncol 19 101 114 2018 29169877 10.1016/S1470-2045(17)30694-0 PMC5847342

[18] Licitra L Mesia R Rivera F et al Evaluation of EGFR gene copy number as a predictive biomarker for the efficacy of cetuximab in combination with chemotherapy in the first-line treatment of recurrent and/or metastatic squamous cell carcinoma of the head and neck: EXTREME study Ann Oncol 22 1078 1087 2011 21048039 10.1093/annonc/mdq588 PMC3082162

[19] Lordick F Kang YK Chung HC et al Capecitabine and cisplatin with or without cetuximab for patients with previously untreated advanced gastric cancer (EXPAND): A randomised, open-label phase 3 trial Lancet Oncol 14 490 499 2013 23594786 10.1016/S1470-2045(13)70102-5

[20] Yang ZY Shen WX Hu XF et al EGFR gene copy number as a predictive biomarker for the treatment of metastatic colorectal cancer with anti-EGFR monoclonal antibodies: A meta-analysis J Hematol Oncol 5 52 2012 22897982 10.1186/1756-8722-5-52 PMC3447654

[21] Misale S Di Nicolantonio F Sartore-Bianchi A et al Resistance to anti-EGFR therapy in colorectal cancer: From heterogeneity to convergent evolution Cancer Discov 4 1269 1280 2014 25293556 10.1158/2159-8290.CD-14-0462

[22] Hiley C de Bruin EC McGranahan N et al Deciphering intratumor heterogeneity and temporal acquisition of driver events to refine precision medicine Genome Biol 15 453 2014 25222836 10.1186/s13059-014-0453-8 PMC4281956

[23] Pectasides E Stachler MD Derks S et al Genomic heterogeneity as a barrier to precision medicine in gastroesophageal adenocarcinoma Cancer Discov 8 37 48 2018 28978556 10.1158/2159-8290.CD-17-0395 PMC5894850

[24] Janku F Kurzrock R Bringing blood-based molecular testing to the clinic Clin Cancer Res 22 5400 5402 2016 27663595 10.1158/1078-0432.CCR-16-1769

[25] Kato S Krishnamurthy N Banks KC et al Utility of genomic analysis in circulating tumor DNA from patients with carcinoma of unknown primary Cancer Res 77 4238 4246 2017 28642281 10.1158/0008-5472.CAN-17-0628 PMC5729906

[26] Lanman RB Mortimer SA Zill OA et al Analytical and clinical validation of a digital sequencing panel for quantitative, highly accurate evaluation of cell-free circulating tumor DNA PLoS One 10 e0140712 2015 26474073 10.1371/journal.pone.0140712 PMC4608804

[27] Riviere P Fanta PT Ikeda S et al The mutational landscape of gastrointestinal malignancies as reflected by circulating tumor DNA Mol Cancer Ther 17 297 305 2018 29133621 10.1158/1535-7163.MCT-17-0360 PMC5752585

[28] Schwaederle M Chattopadhyay R Kato S et al Genomic alterations in circulating tumor DNA from diverse cancer patients identified by next-generation sequencing Cancer Res 77 5419 5427 2017 28807936 10.1158/0008-5472.CAN-17-0885 PMC5626633

[29] Schwaederlé MC Patel SP Husain H et al Utility of genomic assessment of blood-derived circulating tumor DNA (ctDNA) in patients with advanced lung adenocarcinoma Clin Cancer Res 23 5101 5111 2017 28539465 10.1158/1078-0432.CCR-16-2497 PMC5581668

[30] Frampton GM Fichtenholtz A Otto GA et al Development and validation of a clinical cancer genomic profiling test based on massively parallel DNA sequencing Nat Biotechnol 31 1023 1031 2013 24142049 10.1038/nbt.2696 PMC5710001

[31] Tsimberidou AM Iskander NG Hong DS et al Personalized medicine in a phase I clinical trials program: The MD Anderson Cancer Center initiative Clin Cancer Res 18 6373 6383 2012 22966018 10.1158/1078-0432.CCR-12-1627 PMC4454458

[32] Schwaederle M Zhao M Lee JJ et al Association of biomarker-based treatment strategies with response rates and progression-free survival in refractory malignant neoplasms: A meta-analysis JAMA Oncol 2 1452 1459 2016 27273579 10.1001/jamaoncol.2016.2129

[33] Jardim DL Schwaederle M Wei C et al Impact of a biomarker-based strategy on oncology drug development: A meta-analysis of clinical trials leading to FDA approval J Natl Cancer Inst10.1093/jnci/djv253 2015 10.1093/jnci/djv253 PMC4857149 26378224

[34] Schwaederle M Zhao M Lee JJ et al Impact of precision medicine in diverse cancers: A meta-analysis of phase II clinical trials J Clin Oncol 33 3817 3825 2015 26304871 10.1200/JCO.2015.61.5997 PMC4737863

[35] Maron SB Alpert L Kwak HA et al Targeted therapies for targeted populations: Anti-EGFR treatment for EGFR amplified gastroesophageal adenocarcinoma Cancer Discov 8 696 713 2018 29449271 10.1158/2159-8290.CD-17-1260 PMC5984701

[36] Kim ST Banks KC Pectasides E et al Impact of genomic alterations on lapatinib treatment outcome and cell-free genomic landscape during HER2 therapy in HER2+ gastric cancer patients Ann Oncol 29 1037 1048 2018 29409051 10.1093/annonc/mdy034 PMC5913644

[37] Baselga J Bradbury I Eidtmann H et al Lapatinib with trastuzumab for HER2-positive early breast cancer (NeoALTTO): A randomised, open-label, multicentre, phase 3 trial Lancet 379 633 640 2012 Erratum: Lancet 379:616, 2012 22257673 10.1016/S0140-6736(11)61847-3 PMC5705192

[38] Sartore-Bianchi A Trusolino L Martino C et al Dual-targeted therapy with trastuzumab and lapatinib in treatment-refractory, KRAS codon 12/13 wild-type, HER2-positive metastatic colorectal cancer (HERACLES): A proof-of-concept, multicentre, open-label, phase 2 trial Lancet Oncol 17 738 746 2016 27108243 10.1016/S1470-2045(16)00150-9

[39] Falchook GS Naing A Hong DS et al Dual EGFR inhibition in combination with anti-VEGF treatment: A phase I clinical trial in non-small cell lung cancer Oncotarget 4 118 127 2013 23435217 10.18632/oncotarget.763 PMC3702212

[40] Janjigian YY Smit EF Groen HJ et al Dual inhibition of EGFR with afatinib and cetuximab in kinase inhibitor-resistant EGFR-mutant lung cancer with and without T790M mutations Cancer Discov 4 1036 1045 2014 25074459 10.1158/2159-8290.CD-14-0326 PMC4155006

[41] Falchook GS Naing A Wheler JJ et al Dual EGFR inhibition in combination with anti-VEGF treatment in colorectal cancer Oncoscience 1 540 549 2014 10.18632/oncoscience.73 PMC4278330 25594061

[42] Wheler JJ Tsimberidou AM Falchook GS et al Combining erlotinib and cetuximab is associated with activity in patients with non-small cell lung cancer (including squamous cell carcinomas) and wild-type EGFR or resistant mutations Mol Cancer Ther 12 2167 2175 2013 23963360 10.1158/1535-7163.MCT-12-1208 PMC4138057

[43] Wheler J Falchook G Tsimberidou AM et al Revisiting clinical trials using EGFR inhibitor-based regimens in patients with advanced non-small cell lung cancer: a retrospective analysis of an MD Anderson Cancer Center phase I population Oncotarget 4 772 784 2013 23800712 10.18632/oncotarget.1028 PMC3742837

[44] Janku F Huang HJ Angelo LS et al A kinase-independent biological activity for insulin growth factor-1 receptor (IGF-1R): Implications for inhibition of the IGF-1R signal Oncotarget 4 463 473 2013 23531874 10.18632/oncotarget.886 PMC3717308

[45] Weihua Z Tsan R Huang WC et al Survival of cancer cells is maintained by EGFR independent of its kinase activity Cancer Cell 13 385 393 2008 18455122 10.1016/j.ccr.2008.03.015 PMC2413063

[46] Diaz LA Jr Williams RT Wu J et al The molecular evolution of acquired resistance to targeted EGFR blockade in colorectal cancers Nature 486 537 540 2012 22722843 10.1038/nature11219 PMC3436069

[47] Husain H Scur M Murtuza A et al Strategies to overcome bypass mechanisms mediating clinical resistance to EGFR tyrosine kinase inhibition in lung cancer Mol Cancer Ther 16 265 272 2017 28159915 10.1158/1535-7163.MCT-16-0105

[48] Schwaederle M Parker BA Schwab RB et al Precision oncology: The UC San Diego Moores Cancer Center PREDICT experience Mol Cancer Ther 15 743 752 2016 26873727 10.1158/1535-7163.MCT-15-0795

[49] Wheler JJ Janku F Naing A et al Cancer therapy directed by comprehensive genomic profiling: A single center study Cancer Res 76 3690 3701 2016 27197177 10.1158/0008-5472.CAN-15-3043

[50] Oxnard GR Thress KS Alden RS et al Association between plasma genotyping and outcomes of treatment with osimertinib (AZD9291) in advanced non-small-cell lung cancer J Clin Oncol 34 3375 3382 2016 27354477 10.1200/JCO.2016.66.7162 PMC5035123

[51] Abbosh C Birkbak NJ Wilson GA et al Phylogenetic ctDNA analysis depicts early-stage lung cancer evolution Nature 545 446 451 2017 28445469 10.1038/nature22364 PMC5812436

[52] Ramalingam SS Yang JC Lee CK et al Osimertinib as first-line treatment of EGFR mutation-positive advanced non-small-cell lung cancer J Clin Oncol 36 841 849 2018 28841389 10.1200/JCO.2017.74.7576

